# Notes and News

**Published:** 1905-09-16

**Authors:** 


### NOTES AND NEWS.

The report of the New York Commission on Pneumonia has been published. It dwells on the infectious character of the disease.

Up to the present the number of cholera cases in Prussia exceeds 166, of which more than one-third have proved fatal. We hear also of cases at Dantzig and Constantinople.

The late Sir Charles Cunliffe Smith has bequeathed £1,000 to the London Hospital, and the late Mr. E. C. Murray, of Dublin, has bequeathed £1,000 to the Royal City of Dublin Hospital to found two beds in memory of his family. He also left smaller sums to other Irish charities.

Messrs. Knight, Frank and Rutley sold recently a copy of the original pamphlet by William Harvey on the "Discovery of the Circulation of the Blood," published 1628, for £30, a collection of engravings of medical men realised £40. These came from the collection of the late Dr. Thomas Pettigrew.

Professor G. H. F. Nuttall, F.R.S., of Cambridge University, will deliver the opening address of the winter session of the London School of Tropical Medicine in the Lecture Theatre at the school on Wednesday, October 11th at three o'clock. The first students' dinner will be held the same evening at the Gaiety Restaurant, when Sir Ralph Moor, K.C.M.G., formerly High Commissioner of Southern Nigeria, will occupy the chair.

A provincial sessional meeting of the Royal Sanitary Institute will be held in the Council Chamber, Guildhall, York, on Saturday, October 7th, at 11 a.m., and a meeting of the Yorkshire Branch of the Incorporated Society of Medical Officers of Health will be held in conjunction with this meeting. A discussion will take place on "Pure Milk Supply." The discussion will be opened by a paper by Mr. C. W. Sorensen, of the White Rose Dairy Farm, York, and formerly chief dairy expert to the New Zealand Government, entitled "Some Aspects of the Pure Milk Problem—from within."

The Great Central Railway have now 1,334 servants qualified for ambulance work.

Small-pox has broken out at Nuneaton. Thirty patients are in the Isolation Hospital—they are nearly all unvaccinated children under 12 years of age.

The International Congress of Tuberculosis opens at Paris on October 2nd. Reduced railway fares will be charged to those attending. Information can be obtained from the National Association for the Prevention of Tuberculosis, 20 Hanover Square, W.

The International Surgical Society's first Congress will open on September 18th at Brussels. The Secretary-General is Dr. Depage, 79 Avenue Louise, Brussels. He will send invitations, at request, to the various *fêtes* to be held during the Congress.

It is reported that the Brazilian Government is prepared to offer a huge monetary prize for the discovery of the certain cure of consumption. A committee of reference will be formed, and if the proposal is carried into effect, the prize will be open to the whole world.

A Medical Exhibition will be held in the Horticultural Hall, Vincent Square, on October 2 to 6. It has been organised by the *British and Colonial Druggist*. We are informed by the conductors that a large number of medical men have promised attendance.

A Military Medical Congress is to be held at Detroit this month, at which Fleet - Surgeon J. Lloyd Thomas will represent the Medical Department of the Royal Navy, and Lieut.-Col. Rainsford, C.I.E., principal medical officer for Bermuda, that of the Army.

The Secretary of the Prince Alfred Hospital, Sydney, has just left London on his return to Australia, after a tour of inspection of the principal hospitals of England and America. Mr. W. Epps and his chairman, Professor Stewart, have done much to improve the Prince Alfred Hospital, and it is with a view of studying other methods and adopting any that are suitable and desirable that Mr. Epps undertook his pilgrimage. He is a keen, intelligent, and broad-minded man of business, keenly interested in all the aspects of his work.

The Cork Joint Hospital Board have now sufficient money at their disposal to proceed with the establishment of a sanatorium for consumption. A meeting was recently held at which a site was decided upon. The size and form which the sanatorium is to take is not settled. Two remarks were made during the course of the meeting which show the very hopeful view which is held by a large portion of the public in regard to the cure of consumption. The chairman expressed himself not satisfied that the sites mentioned were suitable for curing consumption, and an alderman asked, "Would the patients be cured in three months?"

